# Alcohol-Attributable Fraction of Ischemic Heart Disease Mortality in Russia

**DOI:** 10.1155/2013/287869

**Published:** 2013-07-15

**Authors:** Y. E. Razvodovsky

**Affiliations:** Grodno State Medical University, Gorky street 80, 230008 Grodno, Belarus

## Abstract

*Objective*. The aim of the present study was to estimate the premature ischemic heart disease (IHD) mortality attributable to alcohol abuse in Russia on the basis of aggregate-level data of mortality and alcohol consumption. *Method*. Age-standardized sex-specific male and female IHD mortality data for the period 1980–2005 and data on overall alcohol consumption were analyzed by means of autoregressive integrated moving average (ARIMA) time series analysis. *Results*. The results of the analysis suggest that 41.1% of all male deaths and 30.7% of female deaths from IHD in Russia could be attributed to alcohol. The estimated alcohol-attributable fraction for men ranged from 24.0% (75+ age group) to 62.0% (15–29 age group) and for women from 20.0% (75+ age group) to 64.0% (30–44 age group). *Conclusions*. The outcomes of this study provide indirect support for the hypothesis that the high rate of IHD mortality in Russia may be related to alcohol, as indicated by a close aggregate-level association between number of deaths from IHD and overall alcohol consumption per capita.

## 1. Introduction

Ischemic heart disease (IHD) remains the largest contributor to morbidity and mortality in Europe, accounting for 1.92 million deaths each year [1]. Over the past decades there was a trend towards a decrease in IHD mortality in Western Europe (WE) [2]. In contrast to Western Europe, in many countries of Eastern Europe (EE) IHD mortality continues to increase [2, 3]. Moreover, dramatic increase in IHD mortality is a major cause of the health decline in EE [4]. The increase in deaths from IHD has been especially pronounced among middle-aged men [1]. Several studies have shown that high cardiovascular mortality in EE could not be explained by traditional risk factors identified in the Western countries [5, 6]. This apparent paradox has been a matter of much speculation, but is still poorly understood. 

Some experts have underlined the importance of binge drinking as the main reason for the IHD mortality crisis in Russia [7–11]. The level of alcohol consumption in Russia is among the highest in the world with an annual sales rate about 10 litres of pure alcohol per capita, while independent estimates show a figure as high as 17 litres [12, 13]. The distinctive trait of Russian drinking culture is the preference for binge drinking of vodka, leading to an increase in deaths from alcohol poisoning and cardiovascular diseases [12, 14–17]. 

Evidence of a major effect of binge drinking on Russian IHD mortality pattern comes from both aggregate level analyses and studies of individuals [10, 18–20]. Close aggregate-level association between fatal alcohol poisoning (as a proxy for binge drinking) and IHD mortality rate in Russia has been reported for the period between 1956 and 1998 [21]. In the Novosibirsk cohort study it was shown that binge drinking (consumption of 160 g or more of pure alcohol on a typical occasion) seems to result in increased risk of death from IHD (RR = 1.27; CI: 0.81–1.99) [22]. Collectively this evidence provides additional support for the hypothesis that unfavorable mixture of higher overall level of alcohol consumption and binge drinking pattern is a major risk factor for IHD mortality in Russia. 

The aim of the present study was to estimate the premature IHD mortality attributable to alcohol abuse in Russia using aggregate-level data of IHD mortality and estimates of overall level of alcohol consumption from 1980 to 2005.

## 2. Material and Methods

### 2.1. Data

The data on age-adjusted sex-specific IHD mortality rates per 1000,000 of the population are taken from the Russian State Statistical Committee (*Rosstat*). The Rosstat cause of death classification has undergone several changes in recent decades. Until 1988 the cause of death classification was based upon the Soviet nomenclature which had a limited number of causes of death in comparison with the International Classification of Diseases (ICD) system. From 1989 to 1998 Rosstat used a coding scheme that was based on ICD-9. From 1999 a new coding system based on ICD-10 was introduced. Rosstat issued a table of correspondence between its classification system and ICD-9 and ICD-10, and it has been claimed that the Russian system of coding was and is compatible with the ICD. For example, Soviet classification 90–95 “ischemic heart disease” corresponds with ICD-9 code E 410–E 414 and with ICD-10 code I20–I25. 

The overall level of alcohol consumption in Russia has been estimated using the indirect method based on alcohol psychoses incidence rate and employing ARIMA model. This method is based on the difference between the observed level of alcohol-related harm and level estimated from recorded alcohol consumption [23].

### 2.2. Statistical Analysis

To examine the relation between changes in the alcohol consumption and IHD mortality across the study period a time series analysis was performed using the statistical package “Statistica.” The dependent variables were the annual IHD mortality and the independent variable was aggregate overall alcohol consumption. Bivariate correlations between the raw data from two time series can often be spurious due to common sources in the trends and due to autocorrelation [24]. One way to reduce the risk of obtaining a spurious relation between two variables that have common trends is to remove these trends by means of a “differencing” procedure, as expressed in formula

(1) $$\begin{array}{c} \nabla x_{t}=x_{t}-x_{t-1}. \end{array}$$

This means that the annual changes “∇” in variable “*X*” are analyzed rather than raw data. The process whereby systematic variation within a time series is eliminated before the examination of potential causal relationships is referred to as “prewhitening.” This is subsequently followed by an inspection of the cross-correlation function in order to estimate the association between the two prewhitened time series. It was Box and Jenkins [25] who first proposed this particular method for undertaking a time series analysis, and it is commonly referred to as ARIMA (autoregressive integrated moving average) modeling. We used this model specification to estimate the relationship between the time series IHD mortality and alcohol consumption rates in this paper. In line with previous aggregate studies [24, 26], we estimated semi-logarithmic models with logged output. The following model was estimated:

(2) $$\begin{array}{c} \nabla \text{Ln}M_{t}=a+\beta \nabla A_{t}+\nabla N_{t}, \end{array}$$

where ∇ means that the series is differenced, *M* is IHD mortality rates *a* indicates the possible trend in IHD mortality due to other factors than those included in the model, *A* is the alcohol consumption, *β* is the estimated regression parameter, and *N* is the noise term. The percentage increase in IHD mortality rate associated with a 1-litre increase in alcohol consumption is given by the expression (exp⁡(*β*_1_) − 1)∗100.

In addition to the estimated effect parameter, the alcohol effect will also be expressed in terms of alcohol-attributable fraction (AAF), which is interpreted as the proportion of IHD deaths that are attributable to alcohol. AAF can be calculated from the estimates obtained in ARIMA models according to the following formula: AAF = 1 − exp⁡(−*bX*), where *X* is alcohol consumption for the whole study period and *b* is the estimated effect parameter [27].

## 3. Results

The specification of the bivariate ARIMA model and outcome of the analyses are presented in Table 1. According to the results, alcohol consumption is a statistically significant factor associated with both male and female IHD mortality rates, implying that a 1-litre increase in per capita consumption is associated with an increase in male mortality of 3.9% and female mortality of 2.7%. The estimated effect of alcohol consumption on the age-specific IHD mortality rates for men ranged from 2.0% (75+ age group) to 7.2% (15–19 age group) and for women from 1.6% (75+ age group) to 8.9% (30–44 age group).

A feasible way of estimation of the impact of the alcohol factor on IHD mortality is to compute the alcohol-attributable fraction on the basis of aggregate findings. Table 1 shows the relative proportion of alcohol-attributable deaths to all deaths by gender and age. The results of the analysis suggest that 41.1% of all male deaths and 30.7% female deaths from IHD in Russia could be attributed to alcohol. The estimated AAF for men ranged from 24.0% (75+ age group) to 62.0% (15–29 age group) and for women from 20.0% (75+ age group) to 64.0% (30–44 age group).

## 4. Discussion

According to the results there was a positive and statistically significant effect of per capita alcohol consumption on IHD mortality in Russia. These findings provide support for the binge drinking hypothesis and suggested that episodic heavy drinking of spirits is an important determinant of IHD mortality crisis in Russia.

There is convincing evidence that light to moderate alcohol consumption protects against the risk of IHD, while heavy episodic or binge drinking is associated with an increased risk of cardiovascular events [28, 29]. Studies that have looked at pattern of drinking have consistently found that binge drinking results in an increased risk of IHD death [30–32]. A recent meta-analysis of 14 studies containing 4,718 IHD events suggests that heavy irregular drinking occasions (60 g of pure alcohol per occasion) are significantly associated with incidence of IHD morbidity and mortality compared with regular moderate drinking (RR = 1.45; CI: 1.24–1.7) [33]. This meta-analysis offers evidence that drinking pattern modifies the effects of alcohol intake on the IHD risk. That means that the cardioprotective effect of moderate alcohol consumption disappears when light to moderate drinking is mixed with irregular heavy drinking occasions. Researches have shown that binge drinking produces adverse cardiovascular effects including an atherogenic profile, increased risk of thrombosis and probability of arrhythmia [18, 19]. 

Estimation of the proportion of the alcohol-attributable fraction suggests that 41.1% of male deaths and 30.7% of female deaths from IHD in Russia are attributable to alcohol. The proportion of alcohol-attributable deaths varied widely between age groups, indicating the difference in alcohol consumption rate. As expected, young- and middle-age men and women had the largest proportion of alcohol-attributable deaths with more than half of all deaths attributed to alcohol. This reflects the fact that the level of alcohol-related problems among young and middle aged is especially high.

It should be noted that the estimates of AAF for women, where heavy drinking is restricted to a relatively small proportion of the population, give rise to the suspicion of possible measurement error. It should be recognized that ignoring the confounding variables (unemployment, material deprivation, psychosocial stress, and quality of health care) may imply that the alcohol effect is overestimated, leading to upward bias. Nevertheless, there are some indications that Russian women are drinking more now which is likely to be a factor in the narrowing of the male-female alcohol-related mortality rate ratio [34]. In his recent study, based on the results of RLMS, Perlman highlighted that heavy frequent drinking almost doubled in women between 1994 and 2004 [35]. Furthermore, younger women drank more than their older counterparts, suggesting that more young women are adopting risky lifestyles [35].

Before concluding, several potential limitations of this study must be mentioned. In particular, there was the risk of omitted variable bias in this work. It can be assumed that psychosocial distress and alcohol consumption effects on IHD mortality are spurious indicators of the impact of other powerful risk factors, that is, smoking. Smoking is associated with higher risk of IHD mortality [36]. The high prevalence of smoking among Russian men (about 60%) probably explains the fact of the high male IHD mortality rate compared with the female mortality rate [37]. However, the use of tobacco products was relatively stable during the 1970s–1980s and has fallen substantially in Russia over the 1990s, suggesting that IHD mortality crisis is not a result of a long-term response to smoking trends [12]. Further, in a recent time series analysis it was highlighted that smoking as indicated from sales data did not have a significant impact on IHD in Russia [21]. 

 In conclusion, the outcomes of this study provide indirect support for the hypothesis that the high rate of IHD mortality in Russia may be related to alcohol, as indicated by a close aggregate-level association between number of deaths from IHD and overall alcohol consumption per capita. The findings from the present study have important implications as regards to cardiovascular mortality prevention indicating that a restrictive alcohol policy can be considered as an effective measure of prevention in countries where there is a higher rate of alcohol consumption and detrimental drinking pattern.

## Figures and Tables

**Table 1:** Estimated effects (bivariate ARIMA model) of overall alcohol consumption on IHD mortality rates and estimation of alcohol-attributable fraction (AAF).

Age	Men				Women			
	Model	Estimates	*P*	AAF	Model	Estimates	*P*	AAF
15–29	0, 1, 1*	0.072	0.000	0.620	1, 1, 0	0.055	0.021	0.530
30–44	0, 1, 1	0.059	0.000	0.550	1, 1, 0	0.089	0.000	0.640
45–59	0, 1, 0	0.053	0.000	0.510	1, 1, 0	0.071	0.000	0.620
60–74	0, 1, 0	0.038	0.000	0.400	1, 1, 0	0.031	0.000	0.330
75+	0, 1, 0	0.020	0.024	0.240	1, 1, 0	0.016	0.038	0.200
15–75+	1, 1, 0	0.039	0.000	0.411	1, 1, 0	0.027	0.000	0.307

*The general form of nonseasonal ARIMA model is (*p*, *d*, *q*), where *p* isthe order of the autoregressive parameter, *d* is the order of differencing, and *q* is the order of the moving average parameter. *Q*-test results for residuals are satisfactory in all models.
